# Thirteen New Patients of *PPP2R5D* Gene Mutation and the Fine Profile of Genotype–Phenotype Correlation Unraveling the Pathogenic Mechanism Underlying Macrocephaly Phenotype

**DOI:** 10.3390/children11080897

**Published:** 2024-07-26

**Authors:** Yinmo Jiang, Bingbing Wu, Xi Zhang, Lin Yang, Sujuan Wang, Huiping Li, Shuizhen Zhou, Yanyan Qian, Huijun Wang

**Affiliations:** 1 Center for Molecular Medicine, Pediatrics Research Institute, Children’s Hospital of Fudan University, National Children’s Medical Center, 399 Wanyuan Road, Shanghai 201102, China; 21211240008@m.fudan.edu.cn (Y.J.); 23211240026@m.fudan.edu.cn (X.Z.);; 2 Department of Rehabilitation, Children’s Hospital of Fudan University, Shanghai 201102, China; 3 Department of Child Health Care, Children’s Hospital of Fudan University, National Children’s Medical Center, 399 Wanyuan Road, Shanghai 201102, China; 4 Neurology Department, Children’s Hospital of Fudan University, National Children’s Medical Center, 399 Wanyuan Road, Shanghai 201102, China

**Keywords:** neurodevelopmental disorders, protein phosphatase 2A, Houge–Janssens syndrome 1

## Abstract

Background: Neurodevelopmental disorders (NDDs) are a group of diseases that severely affect the physical and mental health of children. The *PPP2R5D* gene encodes B56δ, the regulatory subunit of protein phosphatase 2A (PP2A). NDDs related to the *PPP2R5D* gene have recently been defined as Houge–Janssens syndrome 1. Methods: Clinical/whole exome sequencing was performed on approximately 3000 patients with NDDs from 2017 to 2023. In vitro experiments were performed to assess the impairment of variants to protein expression and the assembly of PP2A holoenzyme. The genetic information and phenotypes of the reported patients, as well as patients in this study, were summarized, and the genotype–phenotype relationship was analyzed. The probability of pathogenic missense variants in PPP2R5D was predicted using AlphaMissense (AM), and the relationship between certain phenotype and 3D protein structural features were analyzed. Results: Thirteen new patients carrying twelve *PPP2R5D* gene variants were detected, including five novel missense variants and one novel frameshift variant. In vitro experiments revealed that the frameshift variant p.H463Mfs*3 resulted in a ~50 kDa truncated protein with lower expression level. Except for E420K and T536R, other missense variants impaired holoenzyme assembly. Furthermore, we found that pathogenic/likely pathogenic (P/LP) variants that have been reported so far were all missense variants and clustered in three conserved regions, and the likelihood of P/LP mutations located in these conserved regions was extremely high. In addition, the macrocephaly phenotype was related to negatively charged residues involved in substrate recruitment. Conclusions: We reported thirteen new patients with *PPP2R5D* gene variants and expanded the *PPP2R5D* variant spectrum. We confirmed the pathogenicity of novel variants through in vitro experiments. Our findings in genotype–phenotype relationship provide inspiration for genetic counseling and interpretation of variants. We also provide directions for further research on the mechanism of macrocephaly phenotype.

## 1. Introduction

Neurodevelopmental disorders (NDDs) are a group of diseases that severely affect the development of the central nervous system, leading to great impairment in the functions of motor, speech, cognition, and so on. As a member of the phosphoprotein phosphatase family (PPP family), protein phosphatase 2A (PP2A) plays a vital role in neurological development through the regulation of multiple signaling pathways, cell proliferation and cell death, autoimmunity, and other processes [1,2,3,4,5]. Variants in these genes are associated with NDDs and the *PPP2R5D* gene harbored by most patients. The *PPP2R5D* gene is located in the chromosome 6p21.1 and encodes B56δ, the regulatory subunit B, which determines the function and regulation of PP2A. It consists of four distinct families as follows: B, B′, B″ and B‴ [6]. B56δ is a member of the B′ family, which consists of five distinct gene products with more than 80% conserved [3,4,7,8]. According to Verbinnen et al., it has three recurrently mutated conserved regions, including a conserved acid loop involved in substrate binding (CR1), the conserved region downstream of Short Linear interaction Motif (SLiM) binding domain (CR2), and a region that is 100% conserved among the B’ family (CR3) [5]. The regulatory subunit B, together with the catalytic subunit (encoded by *PPP2CA*) and scaffold A (encoded by *PPP2R1A*) together comprise the PP2A holoenzyme [9,10].

In 2015, the *PPP2R5D* gene was first associated with intellectual disability when Fitzgerald et al. reported four patients with *PPP2R5D* gene variants [11]. In the same year, Houge et al. reported 11 patients and proved that *PPP2R5D* gene variants caused disease through a dominant negative effect. These patients shared similar phenotypes, including motor and speech delay, intellectual disability, hypotonia, seizures, and so on [12]. Some patients also presented with the macrocephaly phenotype. Among the 76 patients with *PPP2R5D* gene variants reported by Oyama et al., patients with different variants presented different percentages of the macrocephaly phenotype [13,14]. However, the expression pattern and the mechanism behind this phenotype are still unclear. Recently, E200K and E198K were considered to be related to early-onset parkinsonism [15,16,17,18,19]. At present, the neurodevelopmental disorder caused by *PPP2R5D* gene variants is defined as the Houge–Janssens syndrome 1 (HJS1) in Online Mendelian Inheritance in Man (OMIM:616355). To date, 50 articles have reported about 190 cases [14,20,21]. However, the detailed phenotype–genotype correlation is unclear, and clinical genetic diagnosis is still the major bottleneck. Moreover, there is insufficient knowledge of the macrocephaly phenotype, especially the expression pattern and the mechanism behind it.

In this study, we reported 13 new patients carrying 12 *PPP2R5D* gene variants including five novel missenses, one novel frameshift, and six reported missenses. We expanded the clinical and genetic spectrum and analyzed the genotype–phenotype correlations. We summarized the expression pattern of the macrocephaly phenotype in the reported patients and provided possible research directions for mechanistic investigation.

## 2. Materials and Methods

### 2.1. Subject and Genomic Sequencing

Clinical/whole exome sequencing was performed on approximately 3000 patients with neurodevelopmental disorders from 2017 to 2023. The QIAamp DNA Blood Mini kit (QIAGEN, Beijing, China) was used to extract genomic DNA from the peripheral blood of the patients and their parents following the manufacturer’s instructions. The library was constructed using the Agilent ClearSeq Inherited Disease panel kit and Agilent SureSelect XT Human All Exon V5 kit (Agilent, Santa Clara, CA, USA). Sequencing was performed on an Illumina HiSeq 2500, Illumina HiSeq X10, or Illumina NovaSeq 6000 platform (Illumina, San Diego, CA, USA). Sequencing was conducted following the protocols described in our published work [22,23,24]. The candidate variants detected in patients and their parents were validated by Sanger sequencing on an ABI 3500XL Genetic Analyzer (Applied Biosystems, Shanghai, China).

### 2.2. In Silico Analysis

Sequence data were mapped to the human reference genome (GRCh37/hg19). Variant calling was performed using the Genome Analysis Toolkit Best Practices Pipeline (Version 3.2.2). The allelic frequencies were annotated from gnomAD (http://gnomad-sg.org/, accessed on 12 June 2020), the ExAC database and our in-house database (more than 50k samples), and the variants were evaluated by SIFT (http://sift.jcvi.org/, accessed on 24 January 2017)), PolyPhen2.2 (http://genetics.bwh.harvard.edu/pph2/, accessed on 24 January 2017), MutationTaster (http://www.mutationtaster.org/, accessed on 24 January 2017), CADD (https://cadd.gs.washington.edu/, accessed on 24 January 2017, version 1.6), and REVEL (https://sites.google.com/site/revelgenomics/, accessed on 5 December 2016). Data interpretation followed the American College of Medical Genetics and Genomics, and the Association for Molecular Pathology (ACMG/AMP) guidelines as well as our previously published work [22].

### 2.3. In Vitro Experiments

Plasmids designed to overexpress HA-tagged *PPP2R5D* were purchased from GeneCopoeia^TM^ company (Rockville, MD, USA). We constructed the *PPP2R5D* mutants using the KOD mutagenesis kit (TOYOBO, Tokyo, Japan) according to the manufacturer’s protocol. After the construction, we confirmed the target mutations and excluded the possible introduced mutations through Sanger sequencing. Then, 293T cells were cultured in complete medium supplemented with DMEM (Gibco, Logan, UT, USA), 10% fetal bovine serum (Gibco), and 1% penicillin/streptomycin (Gibco) in 10 cm Corning dishes. A temperature of 37 °C and 5% CO_2_ was achieved in an airtight chamber. Once the cells reached a density of 70–90%, we transiently transfected the plasmids. The transfection reagents used were Lipofectamine3000 and P3000 (Invitrogen, Carlsbad, CA, USA). Assays were performed according to the manufacturer’s recommendations.

Approximately 48 h after transfection, the cells were harvested, and total protein was extracted with a cell lysis buffer (Cell Signaling Technology, #9803S, Marlborough, MA, USA) supplemented with PMSF (Cell Signaling Technology, #8533) and a protein inhibitor cocktail (Cell Signaling Technology, #5870). Later, the proteins were run and separated on 10% SDS page gels (YaMei, #PG122, Shanghai, China). We blocked the PVDF membrane with bull serum albumin (5%) in 1% TBST for an hour after the protein band was transferred. Then, we incubated the membrane with diluted primary antibody at 4 °C overnight. We warmed up the membrane for 30 min at room temperature and rinsed it before we incubated it with secondary antibody. After rinsing, the membrane was prepared for detection with ECL (Thermo, #34580, Waltham, MA, USA). Co immunoprecipitation (Co-IP) was performed as follows: The cells were grown to approximately 70~90% confluence, and we had overexpressed *PPP2R5D* and its mutants. The protein extraction steps were as described earlier. After retaining some cell lysate as input, the remaining lysate was incubated at 4 °C overnight with anti-HA magnetic beads (Bimake, #B26201, Houston, TX, USA). The combined endogenous PPP2R1A and PPP2CA were detected by Western blotting. The primary antibodies are listed as below: PPP2R5D (Abcam, #ab188323, Cambridge, UK), Vinculin (Cell Signaling Technology, #13901), PPP2R1A (NOVUS, #NBP2-19907, St. Louis, MO, USA), and PPP2CA (Cell Signaling Technology, #2308).

### 2.4. Literature Review and Genotype–Phenotype Summary

The genetic information and phenotypes of individuals with *PPP2R5D* gene variants in the HGMD (https://my.qiagendigitalinsights.com//bbp/view/hgmd/pro/all.php/ (assessed on 1 March 2024)) and ClinVar (https://www.ncbi.nlm.nih.gov/clinvar/ (assessed on 1 March 2024)) databases were summarized. The following terms were used in searching PubMed and Web of Science database: “(variants OR mutations) AND (PPP2R5D) AND (Houge–Janssens syndrome 1), a total of 50 publications were carefully checked and the genetic information and phenotypes of individuals were summarized. The duplicate cases were excluded.

### 2.5. AlphaMissense (AM) Prediction and Protein 3D Structure

We provided the observed pathogenic mutations to the trained AM and used the AlphaFold predicted protein 3D features to visualize predicted pathogenicity. Protein 3D features were collected from Uniprot database, Dictionary of Secondary Structure of Protein (DSSP) database [25,26], PDB sum database, and PhosphoSitePlus databse. A two-sided Fisher’s exact test was performed to identify the relationship between certain phenotypes and 3D protein features.

## 3. Result

### 3.1. General Information and Clinical Characteristics

Thirteen patients carrying *PPP2R5D* gene variants, including nine males and four females, were enrolled in this study. Their average age at genetic test was 3 years old (4 days~13 years), and the major complaints of these patients were delayed developmental milestones (69.23%, 9/13), seizures (15.38%, 2/13), and neonatal complications (15.38%, 2/13). Except for patient 3, who had a family history of dwarfism, there was no family history among the other patients. Among them, 77.78% (7/9) presented intellectual disability, 80% (8/10) presented motor delay, and 90% (9/10) presented speech delay. Additionally, 25% (2/8) showed macrocephaly, 20% (2/10) had hypotonia, and 44.44% (4/9) had behavioral problems. Three patients had seizures, where patient 7 presented with spasms, patient 9 had limb shaking, and patient 13 had febrile convulsion. Furthermore, patient 1 had esotropia. Among the nine patients who underwent brain MRI, patient 2 showed an abnormal signal in bilateral basal ganglia, patient 7 showed a high T1WI signal of the globus pallidus, and patient 8 had a slightly wider frontotemporal sulcus (Table 1).

### 3.2. Genetic Findings

**Figure 1:**
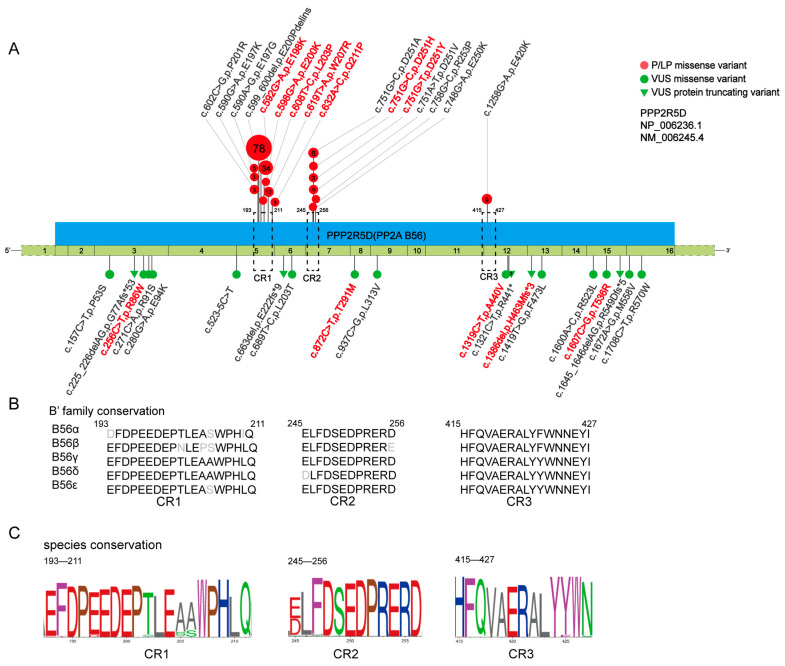
Variant distribution of *PPP2R5D* gene detected in 190 patients and conservation of three conserved regions. (**A**) Location and type of the *PPP2R5D* gene variants. CR1, CR2, and CR3, conserved region 1, 2, and 3. P/LP missense variants are indicated by red circles above the line, VUS missense variants by green circles under the line, VUS protein truncating variants by green triangles. The more patients carrying the variant, the bigger the circles. Patients in our study are shown in bold red. CR1 is from amino acid 193 to 211, CR2 is from 245 to 256 and CR3 is from 415 to 427. PPP2R5D protein is represented by a blue square, the green squares below are exons that encode it. (**B**) Conservation among B’ family of the three conserved regions (CR1–CR3). (**C**) Evolutionary conservation of the three conserved regions.

Twelve variants of the *PPP2R5D* gene were detected, including five novel missense variants (L203P, T291M, A440V, R86W and T536R), one novel frameshift variant (H463Mfs*3), and six reported missense variants (E198K, E200K, W207R, Q211P, D251H and D251Y). E198K, W207R, D251Y, and L203P were confirmed to be de novo (Supplementary Figure S1). We classified E198K, E200K, W207R, Q211P, D251H, and D251Y as pathogenic (P) variants, and L203P as a likely pathogenic (LP) variant. Other variants were classified as variants of uncertain significance (VUS) based on the available genetic evidence. Variants E198K, E200K, L203P, W207R, and Q211P were located in CR1, while D251H and D251Y located in CR2. The remaining five variants were scattered outside the three conserved regions of PPP2R5D (Table 1, Figure 1A).

### 3.3. Genotype–Phenotype Correlation Analysis Shown Hot Spots and Core Region of PPP2R5D Gene Variants

A total of 190 patients sharing 35 variants of the *PPP2R5D* gene were reviewed from publications, ClinVar, the HGMD online database, and our study. Among these variants, there were twenty-eight variants that were missense, five were frameshift, one was nonsense, and one was splicing. We re-classified them according to the ACMG guideline. For variant p.R441*, we reclassified it from LP to VUS, and for variant p.P53S, we reclassified it from VUS to benign (Supplementary Table S1). In total, there were 17 P/LP variants, 17 VUS variants, and 1 benign variant. The 17 P/LP variants were all missense variants and were located in three conserved regions of PPP2R5D. Among them, ten variants were located in CR1, six in CR2, and one in CR3 (Figure 1A). These three regions showed high conservation across B’ family members and different species (Figure 1B,C). We calculated the distance between variants in unit of base pairs according to Lelieveld et al. [27]. The average distance of variants in CR1 was 5.67 base pairs, while in CR2, it was 2.33 base pairs. Therefore, most of the variants were in CR1, while the variants in CR2 were more concentrated, narrowed down to 2.33 base pairs (Supplementary Table S1).

Among the VUS variants, ten were missenses, six were frameshifts, and one was splicing. These VUSs were scattered outside the three conserved regions (Supplementary Table S1).

There were 169 patients with P/LP variants, and the common clinical features among them were intellectual disability (100%, 135/135). However, the percentage of macrocephaly (83.7%, 77/92) and epilepsy (53.93%, 48/89) varied, even within the same variant. For example, among the 78 patients with E198K, 88.24% (45/51) of them presented macrocephaly, and 60% (30/50) presented epilepsy. Among the 34 patients with E200K, 66.67% (8/12) had macrocephaly, and only 13% (2/15) had epilepsy. Of the nine patients with E420K, all four of them (100%,4/4) showed macrocephaly, while none of them (0%, 0/4) had epilepsy (Supplementary Table S2).

### 3.4. In Vitro Functional Assay

**Figure 2:**
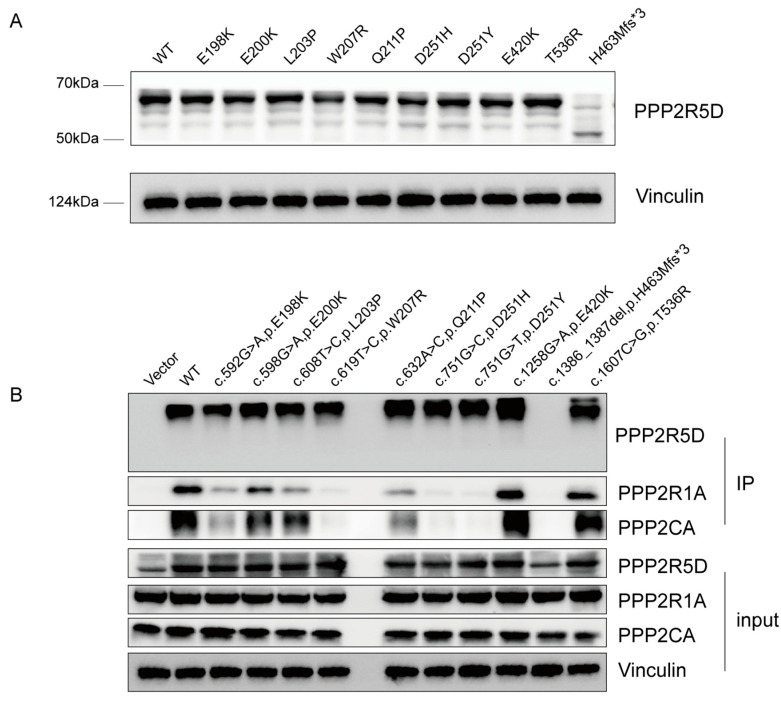
Protein expression levels of PPP2R5D and its interaction with the PPP2CA and PPP2R1A. (**A**) HA-tagged PPP2R5D WT/variants were transfected in 293T cells, protein expression was detected through Western blot. A 66 kDa band was detected in all missense variants. As for frameshift variant H436Mfs*3, a ~50 kDa band was detected and it was significantly weaker. (**B**) HA-tagged PPP2R5D WT/variants were purified through HA pull down, the interaction with endogenous PPP2CA and PPP2R1A were detected through Western blot. No band of PPP2CA and PPP2R1A was detected in W207R, D251H, D251Y and H463Mfs*3. A weaker band of PPP2CA and PPP2R1A was detected in E198K, E200K, L203P and Q211P.

We constructed HA-tagged expression plasmids of E198K, E200K, L203P, W207R, Q211P, D251H, D251Y, T536R, E420K, and H463Mfs*3. These plasmids were transiently transfected into 293T cells. Western blot results showed that the frameshift variant H463Mfs*3 escaped the nonsense-mediated decay (NMD) and resulted in a ~50 kDa truncated protein with a lower expression level. The nine missense variants did not alter protein expression (Figure 2A). The Co-IP results indicated that the binding of mutants to endogenous PPP2R1A and PPP2CA was significantly impaired to varying degrees. PPP2R5D^W207R^, PPP2R5D^D251H^, PPP2R5D^D251Y^, and PPP2R5D^H463Mfs*3^ barely interacted, while PPP2R5D^E198K^ and PPP2R5D^Q211P^ interacted in small amounts. PPP2R5D^L203P^ and PPP2R5D^200K^ had the least interaction but also the significant effect on the binding capacity (Figure 2B). However, PPP2R5D^T536R^ and PPP2R5D^E420K^, due to the endogenous A, C subunits, did not significantly interact.

### 3.5. AM Pathogenicity Prediction and Structural Effects of Missense Variants on Phenotypes

**Figure 3:**
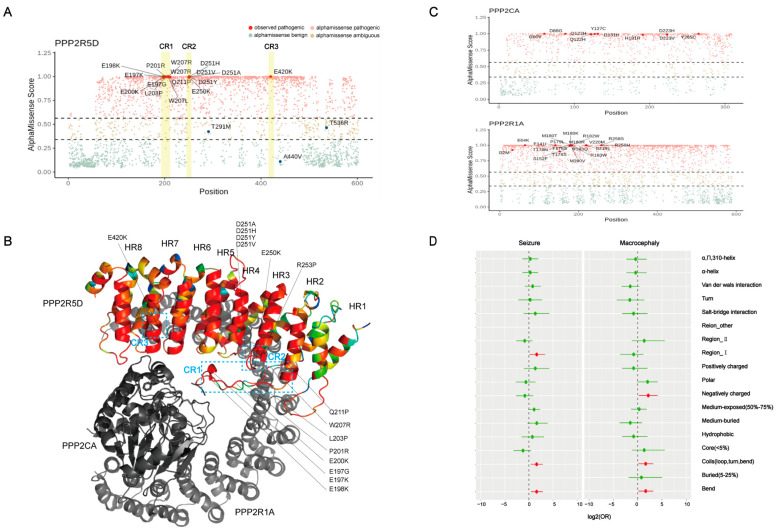
Pathogenicity prediction of PPP2R5D and analysis of residue characterization and phenotype. (**A**) Pathogenicity prediction of PPP2R5D by AM, the yellow bar represents conserved region 1, 2, and 3. Observed pathogenic variants were represented by red dots. (**B**) Protein 3D structure of PP2A holoenzyme. PPP2R5D protein was colored by scores predicted by AM, from green to red, low to high. PPP2R1A and PPP2CA were colored grey and darker gray. Amino acids affected by pathogenic missense variants of *PPP2R5D* gene were labeled on the protein. (**C**) Pathogenicity prediction by AM of PPP2CA and PPP2R1A. (**D**) Two-sided Fisher’s exact test of phenotypes and characterization of residues. Circles represent the OR, and the horizontal bars represent 95% CI. Red circles mean that the features in the y axis were enriched in patients with the phenotype, green means the opposite.

We used AM to predict the variants of PP2A subunit encoding genes and categorized them as “AM pathogenic”, “AM ambiguous”, and “AM benign” based on the predicted scores. We observed that the AM score of 17 pathogenic missense variants in *PPP2R5D* gene were close to a high score of 1. Interestingly, we found that all the predicted missense variants located in the three conserved regions were given an extremely high likelihood of being pathogenic (Figure 3A). Then, we color-coded the amino acid of the PPP2R5D protein according to the pathogenicity prediction by AM. We noticed a continuous red color in the α-helix of HEAT-repeat 3~8. However, currently, no pathogenic variants have been reported in these regions (Figure 3B). Additionally, we also observed that variants of *PPP2R1A* gene showed spatial clustering in HEAT-repeat 4~6 and 12, which is consistent with our previous study [28] (Figure 3C).

Additionally, we analyzed the association between these phenotypes and characterization of substituted residues. The macrocephaly phenotype was specifically associated with negatively charged residues. Seizures specifically related to residues participated in conserved region 1. Both phenotypes were related to residues involved in protein secondary structures such as coils and bends (Figure 3D).

## 4. Discussion

The *PPP2R5D* gene encodes B56δ, a member of the B′ family involved in the formation of variable regulatory subunit B. The regulatory subunit assembles with the catalytic subunit and scaffold subunit to form the PP2A holoenzyme. The B′ family consists of α, β, γ, δ, and ɛ, encoded by *PPP2R5A*, *PPP2R5B*, *PPP2R5C*, *PPP2R5D*, and *PPP2R5E* genes, respectively. Currently, most of the P/LP variants were harbored by the *PPP2R5D* gene, although variants in *PPP2R5C* and *PPP2R5E* have also been identified in the ClinVar database.

In this study, we reported thirteen new patients with *PPP2R5D* gene variants [14,15,16,17,18,19,21]. Our results suggest that missense variants located in CR1 and CR2 lead to impaired binding of B56δ to endogenous subunit A and C, while the protein expression remained unchanged. Since the *PPP2R5D* gene variants cause disease through a dominant-negative effect [11], we classified the novel variant L203P as LP, and the other four novel missense variants as VUS. The frameshift variant p.H463Mfs*3 escaped nonsense-mediated decay and produced a ~50 kDa truncated protein. The expression level and binding ability to A, C subunits was significantly reduced. However, there is no current evidence that these variants lead to NDDs through a loss-of-function mechanism. Therefore, we ultimately rated variant p.H463Mfs*3 as VUS as per the ACMG guideline.

We also summarized data from 190 reported patients. We found that all the P/LP variants of the *PPP2R5D* gene reported so far were missense variants located in the three conserved regions (CR1, CR2 and CR3). Using the AM method, we identified four predicted regions in PPP2R5D with a high likelihood of P/LP variants. These four predicted regions were located in the alpha helix of HEAT-repeat 4, 5, 6, and 8. Their amino acid sequences were from 295 to 316, from 341 to 353, from 373 to 389, and from 457 to 475, respectively. These four regions were highly conserved among B56 family (Supplementary Figure S2). Interestingly, no P/LP variants were reported in these regions. Although CR1, CR2, and CR3 are spatially separated, they are harbored by a super-long regulatory interface of PP2A, and P/LP variants on these three conserved regions can globally alter the interface [29]. The four regions predicted by us were not involved in forming the regulation interface, which may explain why no pathogenic variants have been reported in them. The AM method predicts the incidence of P/LP variants based on the base pair and 3D protein structure of the reference sequence and characteristics of human proteins [30]. For B56δ, the residues involved in the spatial protein–protein interaction are more important and determine their actual pathogenicity. Therefore, AM prediction provides only one type of evidence for the probability of pathogenicity.

We also found that no P/LP frameshift variants have been reported so far. In the gnomAD database, 22 frameshift variants were found in 40 normal individuals. The size of the protein products of these frameshift variants ranged from 85 amino acids to 651 amino acids. Clinical information on carriers of the variants marked by red triangles can be found in UKBiobank, and they did not present clinical manifestations associated with neurodevelopmental disorders (Supplementary Figure S3). In a three-generation pedigree with the nonsense variant of p.Arg441*, one family member with this variant was phenotypically healthy [31]. In vitro experiments showed that in B56δ truncated without C-terminal, PP2A lost its auto-inhibition ability, increased the phosphatase activity, and the binding of substrate SLiMs. It is possible that the N-arm stabilizes the binding stage of the C-arm by the suppressing the active site but causes structural tension toward the SLiM-binding groove [29].This may explain the absence of phenotypes in individuals with C terminal truncated variants.

In this study, we found that 83.7% of patients had macrocephaly. Interestingly, the percentage of macrocephaly differed among the different variants. PPP2R5D formed a complex with AKT and participated in the mTOR pathway activity thus regulating cell growth [32]. It is highly possible that the incidence of macrocephaly is determined by the enzyme activity change caused by gene mutations and the way mTOR pathway is activated. For example, all E420K patients with recorded head circumstances presented macrocephaly. Researchers have discovered that the variant E198K activates the mTOR pathway only through ERK, whereas E420K activates the mTOR pathway through both ERK and AKT [32,33]. Additionally, we found that the glutamate was substituted with lysine in nearly 90% (49/55) of the macrocephaly patients. Through the analysis of residue characteristics and phenotypes, we found that macrocephaly is associated with substitutions of residues that are negatively charged. B56δ recruits positively charged substrates through a negatively charged acidic surface [34]. When a negatively charged residue on the surface is substituted, it results in a loss of negative charge, ultimately leading to a decrease in the dephosphorylation efficiency of PP2A. Since PP2A combines with AKT and regulates the mTOR pathway by dephosphorylating it, the mTOR pathway is activated when there is a shortage of negative charge [34,35,36]. The E420K variant, with a negatively charged glutamic acid substituted by a positively charged lysine, leads to overactive AKT–mTOR signaling and larger cell size [32]. Generally speaking, different variants affect the enzyme activity and activate mTOR pathway in different ways. More in-depth studies on important functional structural domains, the charge changes, and the conformational changes can provide an explanation for the mechanism behind macrocephaly.

## 5. Conclusions

We confirmed the pathogenicity of novel variants through in vitro experiments, reviewed the patients’ genetic information and clinical phenotypes, summarized the expression pattern of macrocephaly and seizure phenotype. We also predicted the pathogenicity of PPP2R5D and analyzed the relationship between phenotypes and protein features. This provides inspiration for genetic counseling and points the way for research on the mechanisms of the macrocephaly phenotype.

## Figures and Tables

**Table 1:** Clinical information and genetic findings of thirteen patients in this study.

Patient ID	Patient 1	Patient 2	Patient 3	Patient 4	Patient 5	Patient 6	Patient 7	Patient 8	Patient 9	Patient 10	Patient 11	Patient 12	Patient 13
**General information**													
Gender/age	M/3y	F/13y	F/11y	F/5y5m	F/2y1m	M/10m	M/20d	M/3m	M/4d	M/3y	M/3y6m	M/2y7m	M/6y8m
Birth condition	LGA	-	-	Premature birth, SGA	-	-	-	-	-	-	-	+, fetal distress	-
Variants (NM_006245.4)	c.592G>A, p.E198K	c.598G>A, p.E200K	c.598G>A, p.E200K	c.608T>C, p.L203P	c.619T>C, p.W207R	c.632A>C, p.Q211P	c.751G>C, p.D251H	c.751G>T, p.D251Y	c.872C>T, p.T291M	c.1319C>T, p.A440V	c.1386del, p.M463Mfs*3	c.256C>T, p.R86W	c.1607C>G, p.T536R
Conserved Region	Region 1	Region 1	Region 1	Region 1	Region 1	Region 1	Region 2	Region 2	Others	Others	Others	Others	Others
Inheritance	De novo	NA	NA	De novo	De novo	NA	NA	De novo	NA	NA	NA	NA	NA
Class	P	P	P	LP	P	P	P	P	VUS	VUS	VUS	VUS	VUS
**Phenotypes**													
Motor delay	+	+	+	+	+	+	NA	+	NA	-	+	NA	-
Speech delay	+, no words	+	+	+, speak at 2 years old	+	+	NA	NA	NA	+	+	+	-
ID	+	+	+	+	+	+	NA	+	NA	NA	-	NA	-
Macrocephaly	+	-	-	NA	+	NA	NA	-	NA	NA	-	-	-
Hypotonia	+	-	NA	+	-	+	NA	-	-	-	-	-	-
Brain MRI	-	+, abnormal signal in bilateral basal ganglia	NA	-	-	NA	+, high T1WI signal of the globus pallidus	+, Slightly wider frontotemporal sulcus	NA	-	-	NA	+, small cyst in the right parietal lobe
Behavioral problems	-	+	NA	+	-	NA	NA	-	NA	ASD	-	ASD	-
seizures	-	-	-	-	-	-	+	-	+	-	-	-	+
Others	+, esotropia	-	+, short statue	-	-	-	-	-	-	-	-	-	-

Note: F, female; M, male; y, year, m, month, d, day; +, the phenotype is present; -, the phenotype is not present; P, pathogenic; LP, likely pathogenic; VUS, variants of uncertain significance; SGA, small for gestational age; LGA, large for gestational age; NA, not available; ID, intellectual disability; MRI, Magnetic Resonance Imaging.

## Data Availability

Variants detected in this study were submitted to ClinVar with submission number SUB14492438. The data presented in this study are available on request from the corresponding author due to ethical restrictions.

## Supplementary Materials

The following supporting information can be downloaded at: https://www.mdpi.com/article/10.3390/children11080897/s1, Figure S1. Pedigree charts, Sanger sequencing results of E198K, W207R, D251Y and L203P. Figure S2. The predicted four regions with high probability of P/LP missense variants by AM and the conservation of them in B56 family. (A) The 3D structure and HEAT-repeats model of PPP2R5D and the location of four predicted regions. The predicted regions are colored in red. The first region is from amino acids 295 to 316. The second is from 341 to 353. The third is from 373 to 389 and the forth is from 457 to 475. (B) The conservation of B56 family. Figure S3. Frameshift variants and their protein products in gnomAD database. The blue rectangle represents the full length of PPP2R5D. CR1, CR2 and CR3 are indicated by red dashed boxes. The number in the brackets after the frameshift variant are the number of individuals carrying it. Protein products are represented by yellow rectangles, the length of those rectangles represent the size of protein products. Table S1. Thirty five *PPP2R5D* gene variants in 190 patients. Table S2: Clinical information of 169 patients carrying pathogenic/likely pathogenic *PPP2R5D* gene variants.
