# Supplementary material for: Thirteen New Patients of PPP2R5D Gene Mutation and the Fine Profile of Genotype–Phenotype Correlation Unraveling the Pathogenic Mechanism Underlying Macrocephaly Phenotype
Source: Children (Basel). 2024 Jul 26;11(8):897. doi: 10.3390/children11080897 (PMC11352527; doi:10.3390/children11080897)
Supplement: Supplementary file 1 [file children-11-00897-s001.zip › supp figure legend.pdf]

Supplementary Figure S1 Pedigree charts, Sanger sequencing results of E198K, W207R, D251Y and L203P.

Supplementary Figure S2 The predicted four regions with high probability of P/LP missense variants by AM and the conservation of them in B56 family. (A)The 3D structure and HEAT-repeats model of PPP2R5D and the location of four predicted regions. The predicted regions are colored in red. The first region is from amino acids 295 to 316. The second is from 341 to 353. The third is from 373 to 389 and the forth is from 457 to 475. (B)The conservation of B56 family.

Supplementary Figure S3 Frameshift variants and their protein products in gnomAD database. The blue rectangle represents the full length of PPP2R5D. CR1, CR2 and CR3 are indicated by red dashed boxes. The number in the brackets after the frameshift variant are the number of individuals carrying it. Protein products are represented by yellow rectangles, the length of those rectangles represent the size of protein products.
