# Supplementary material for: Thirteen New Patients of PPP2R5D Gene Mutation and the Fine Profile of Genotype–Phenotype Correlation Unraveling the Pathogenic Mechanism Underlying Macrocephaly Phenotype
Source: Children (Basel). 2024 Jul 26;11(8):897. doi: 10.3390/children11080897 (PMC11352527; doi:10.3390/children11080897)
Supplement: Supplementary file 1 [file children-11-00897-s001.zip › Supplementary Figure S2.pdf]

A

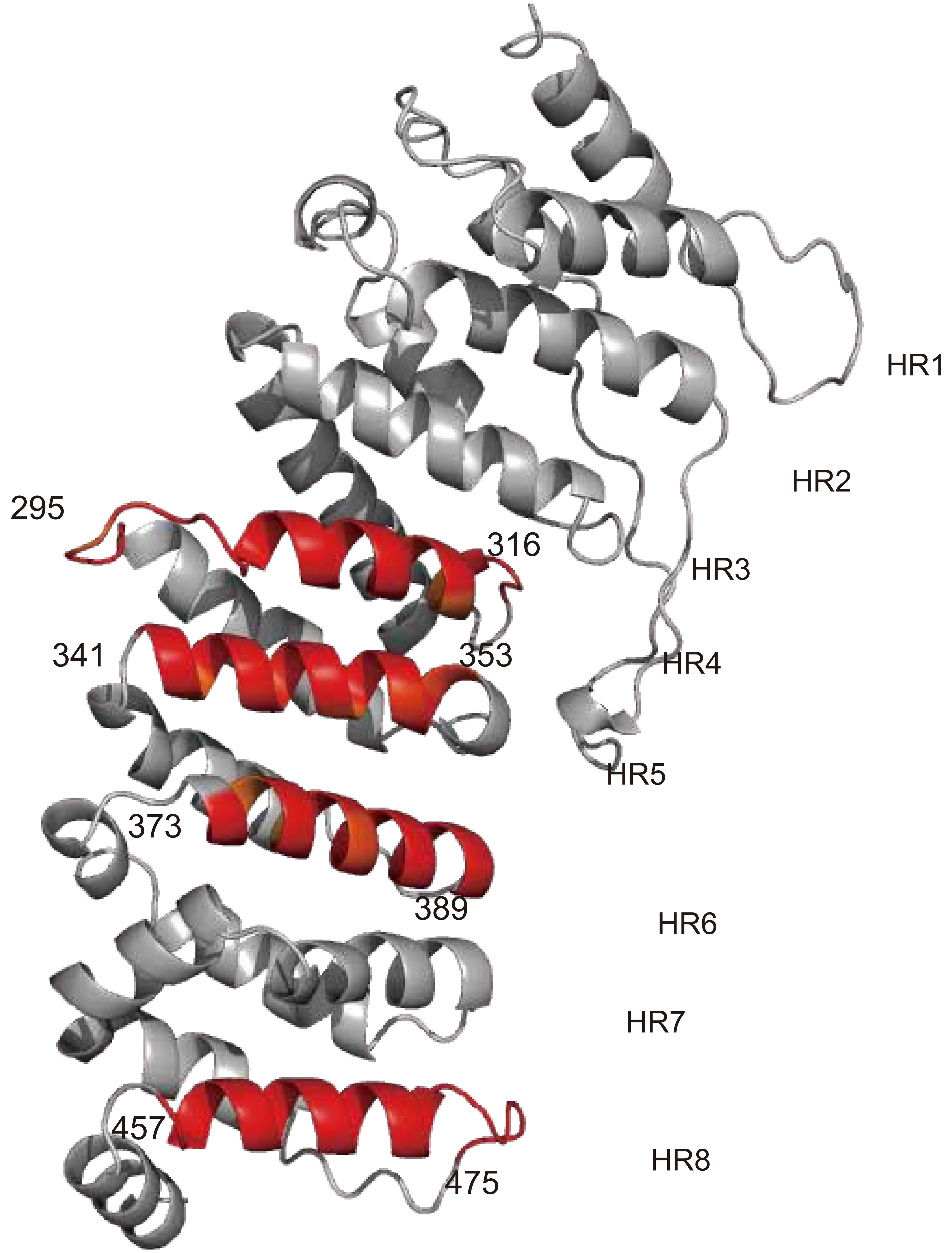

B

	394 … 316	341 … 353
B56α	NGVAELLEILGSIINGFALPLK........	AQLAYCVVQFLEK
B56β	NGVAELLEILGSIINGFALPLK........	AQLAYCVVQFLEK
B56γ	NGIAELLEILGSIINGFALPLK.........	PQLAYCVVQFLEK
B56δ	NGIAELLEILGSIINGFALPLK.........	PQLAYCVVQFLEK
B56ε	NGVAELLEILGSIINGFALPLK........	AQLAYCIVQFLEK

	373 … 389	457 … 475
B56α	CSQKEVMFLGEIEEILD........	HWNPTIVGLIYNVLKTFME
B56β	CSQKEVMFLGEMEEILD......	HWNQTIVGLIYNVLKTFME
B56γ	HSPKEVMFLNELEEILD........	HWNKTIHGLIYNALKLFME
B56δ	HSPKEVMFLNELEEILD........	HWNKTIHGLIYNALKLFME
B56ε	CSQKEVMFLGELEEILD........	HWNPAIVGLIYNVLKAFME
