# Supplementary material for: Thirteen New Patients of PPP2R5D Gene Mutation and the Fine Profile of Genotype–Phenotype Correlation Unraveling the Pathogenic Mechanism Underlying Macrocephaly Phenotype
Source: Children (Basel). 2024 Jul 26;11(8):897. doi: 10.3390/children11080897 (PMC11352527; doi:10.3390/children11080897)
Supplement: Supplementary file 1 [file children-11-00897-s001.zip › supplementary Table S2.pdf]

Supplementary Table S2: Clinical information of 169 patients carrying pathogenic/likely pathogenic *PPP2R5D* gene variants

Variant	Classification	Patient number	Hypotonia	Macrocephaly	Epilepsy
c.592G>A,p.E198K	P	78	19(95%)	45(88.24%)	30(60%)
c.598G>A,p.E200K	P	34	5(100%)	8(66.67%)	2(13%)
c.602C>G,p.P201R	P	3	1(100%)	0(0%)	1(100%)
c.619T>C,p.W207R	P	12	2(66.67%)	4(57.14%)	4(57.14%)
c.589G>A,p.E197K	P	3	1(100%)	2(66.67%)	0(0%)
c.590A>G,p.E197G	P	5	NA	1(50%)	3(100%)
c.620G>T,p.W207L	P	1	1(100%)	1(100%)	NA
c.632A>C,p.Q211P	P	3	1(100%)	1(50%)	2(66.67%)
c.599_602delAGGCinsGGCA,p.G200_P201delinsGH	LP	1	NA	1(100%)	0(0%)
c.608T>C,p.L203P	P	1	1(100%)	NA	0(0%)
c.748G>A,p.E250K	P	2	0(0%)	1(100%)	NA
c.752A>C,p.D251A	P	6	NA	3(100%)	0(0%)
c.752A>T,p.D251V	P	5	NA	4(100%)	2(40%)
c.751G>C,p.D251H	P	2	NA	1(100%)	1(50%)
c.751G>T,p.D251Y	P	3	0(0%)	1(50%)	1(50%)
c.758G>C,p.D253P	P	1	NA	NA	1(100%)
c.1258G>A,p.E420K	P	9	3(100%)	4(100%)	0(0%)

Note: P, pathogenic; LP, likely pathogenic; NA, not available
