# Supplementary figures and images for: Thirteen New Patients of PPP2R5D Gene Mutation and the Fine Profile of Genotype–Phenotype Correlation Unraveling the Pathogenic Mechanism Underlying Macrocephaly Phenotype
Source: Children (Basel). 2024 Jul 26;11(8):897. doi: 10.3390/children11080897 (PMC11352527; doi:10.3390/children11080897)

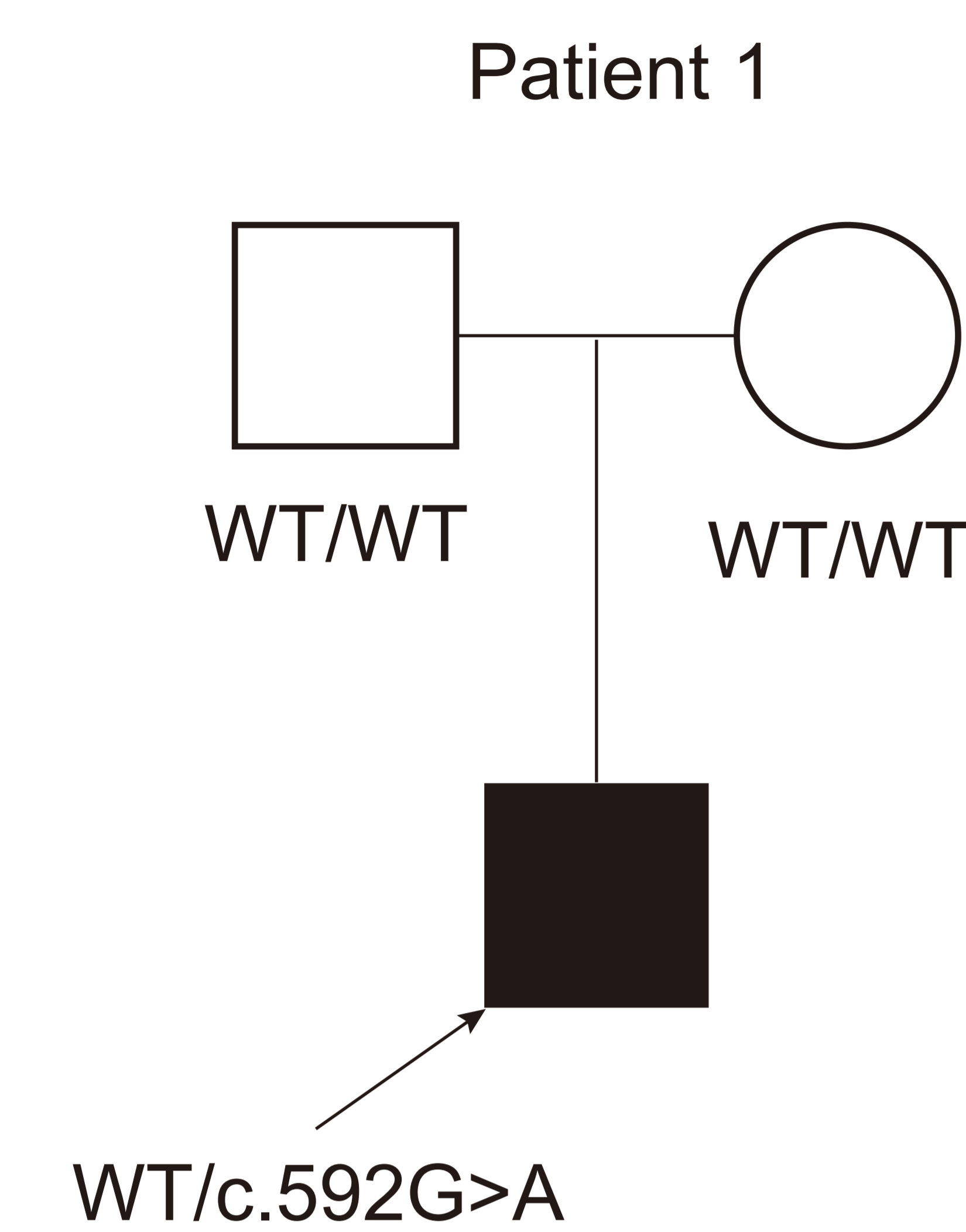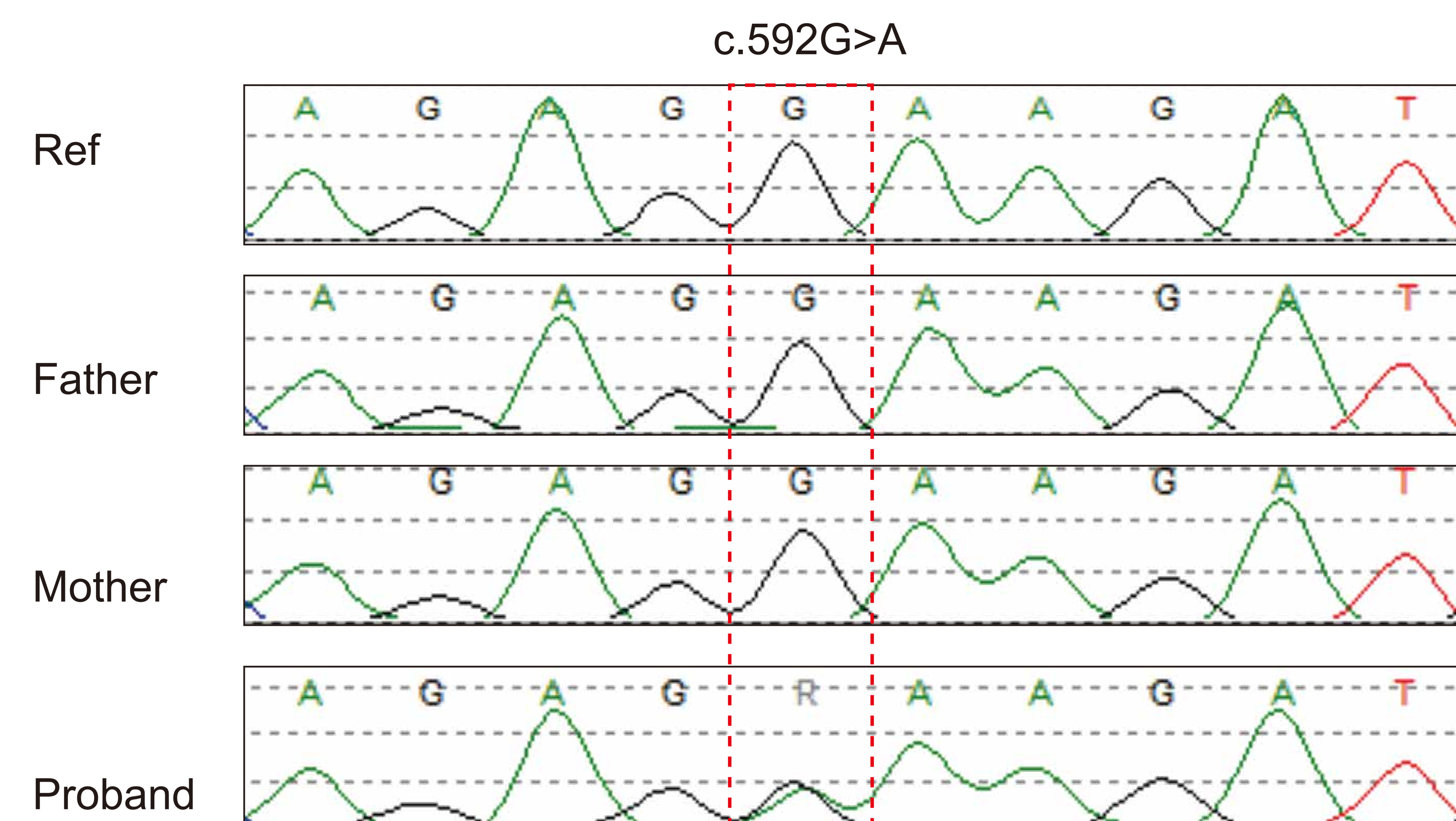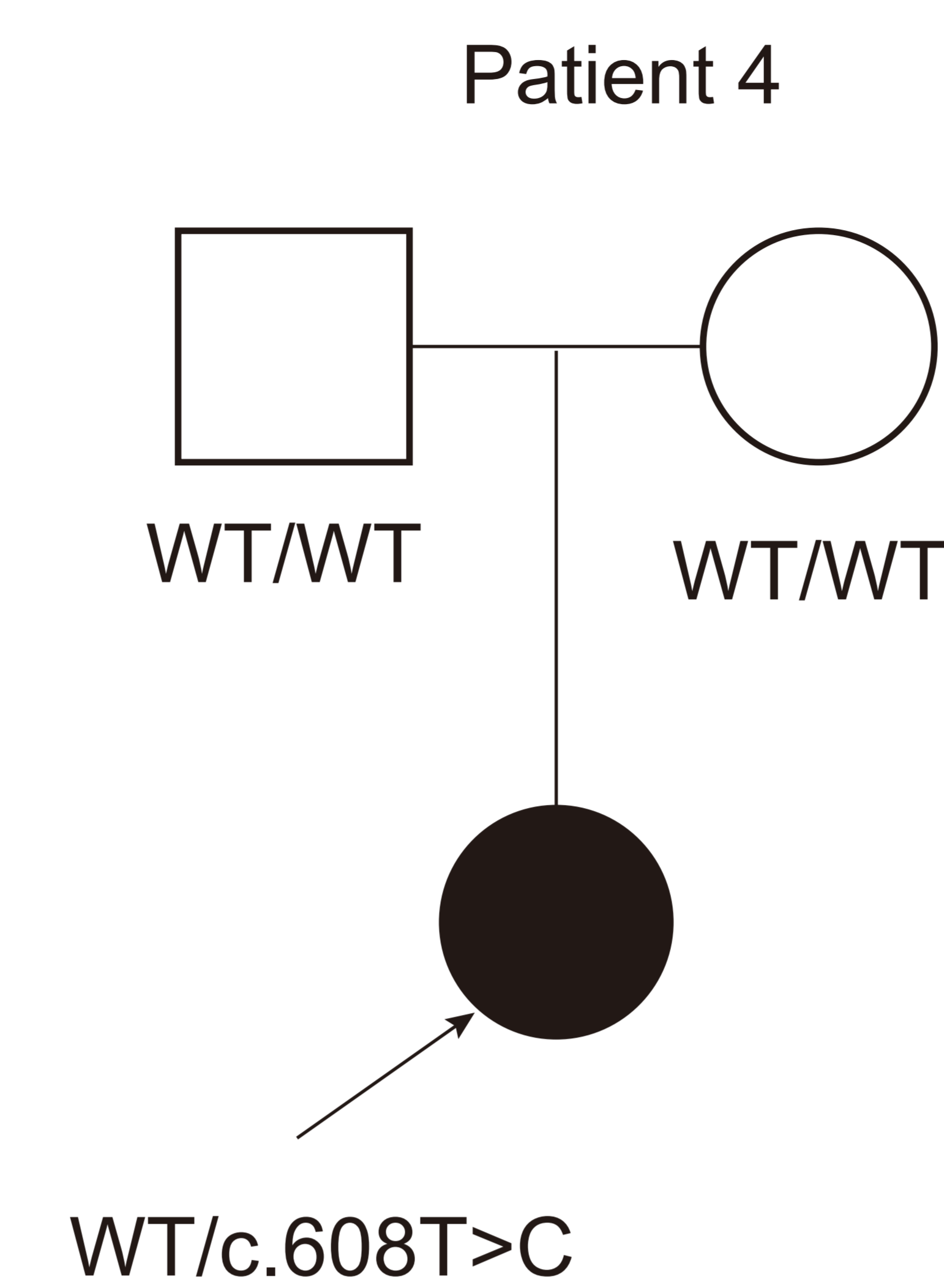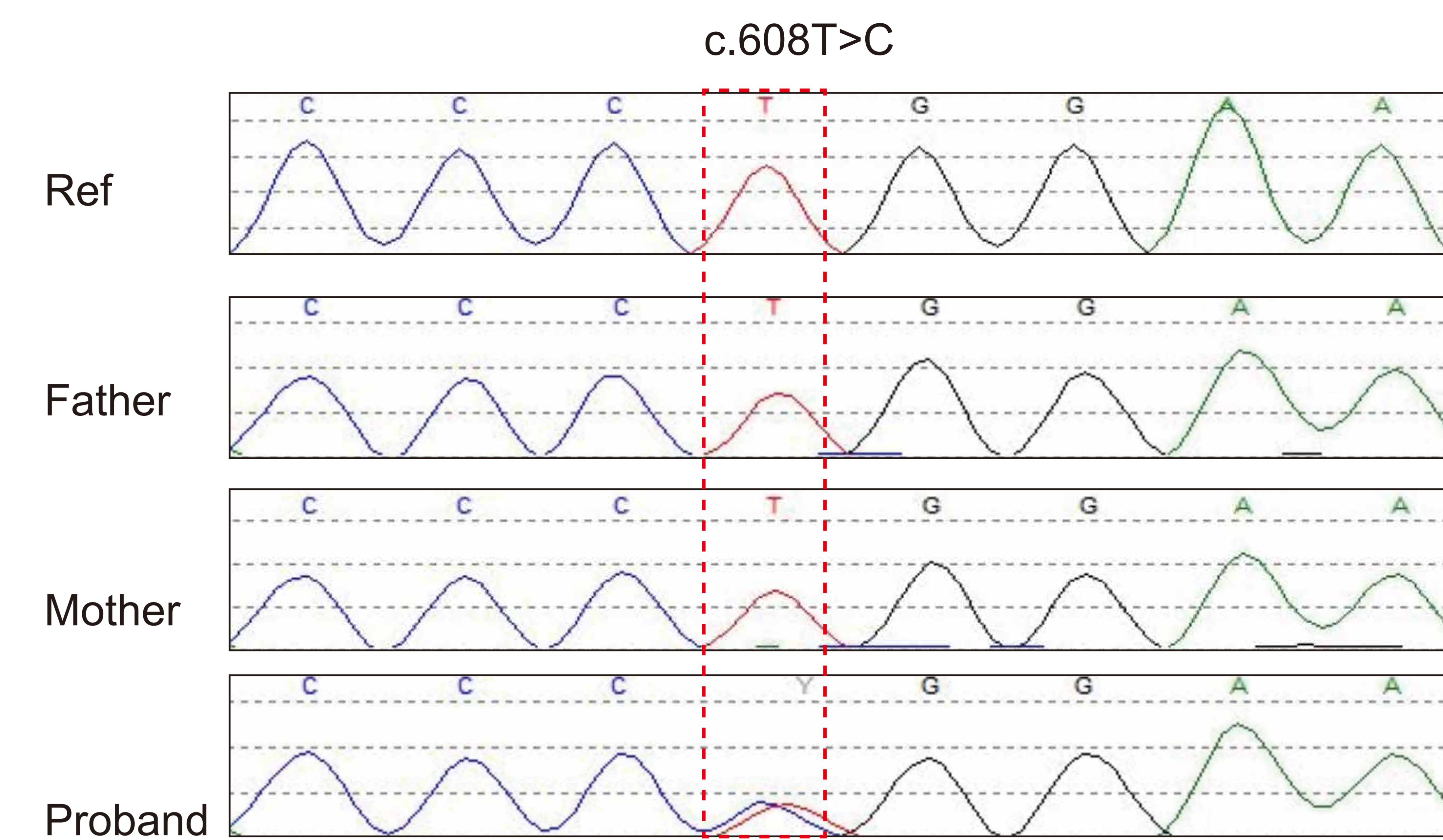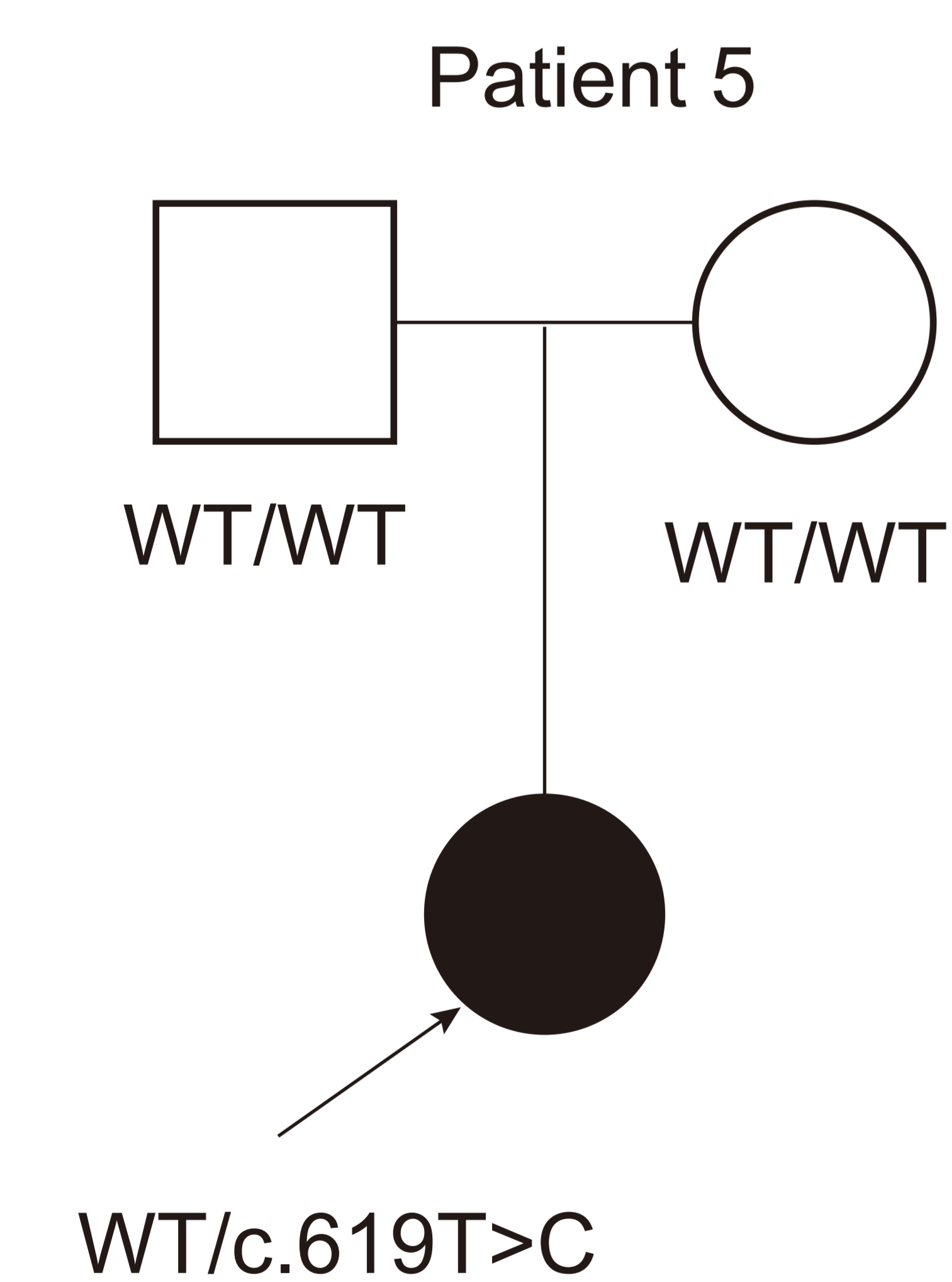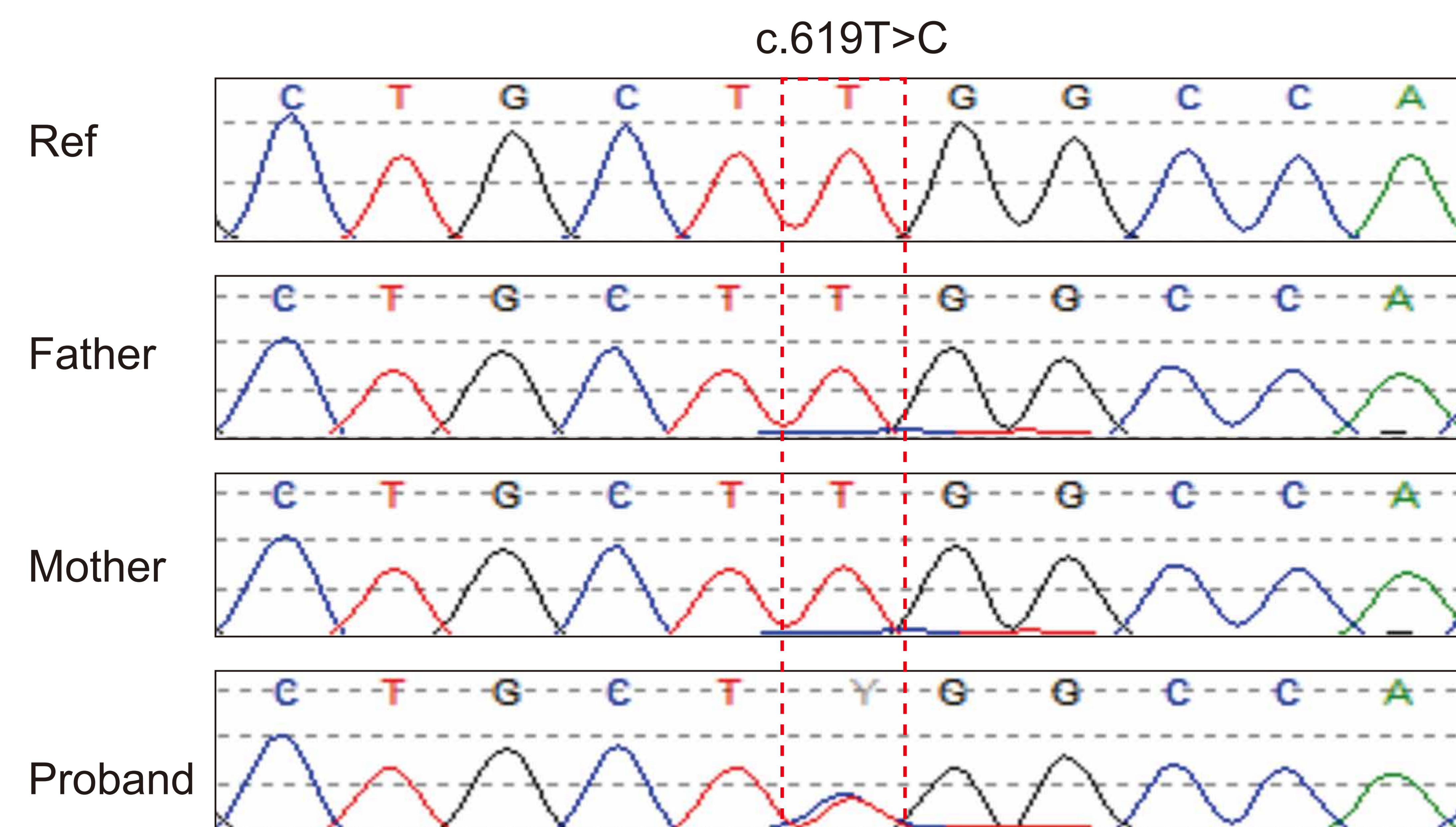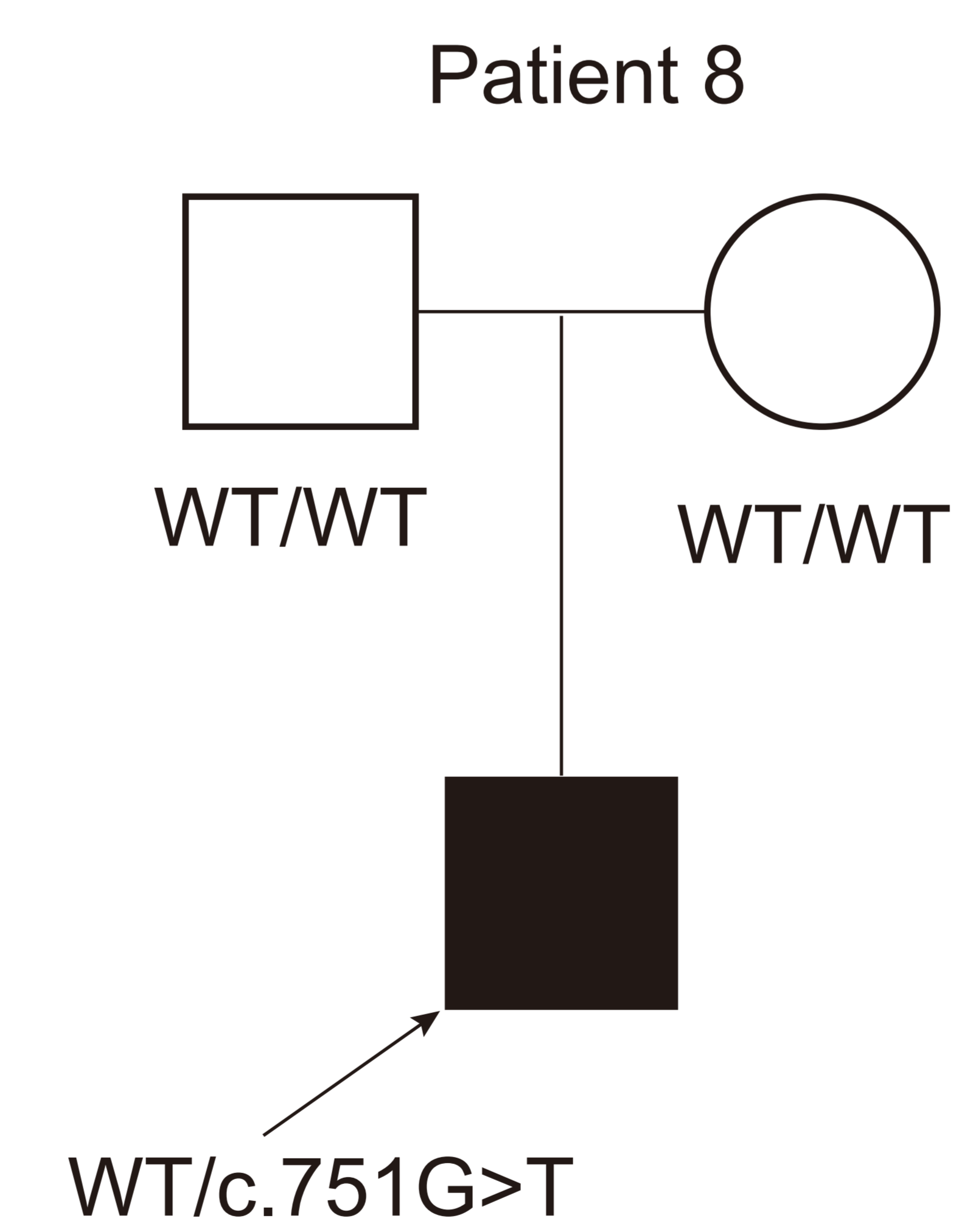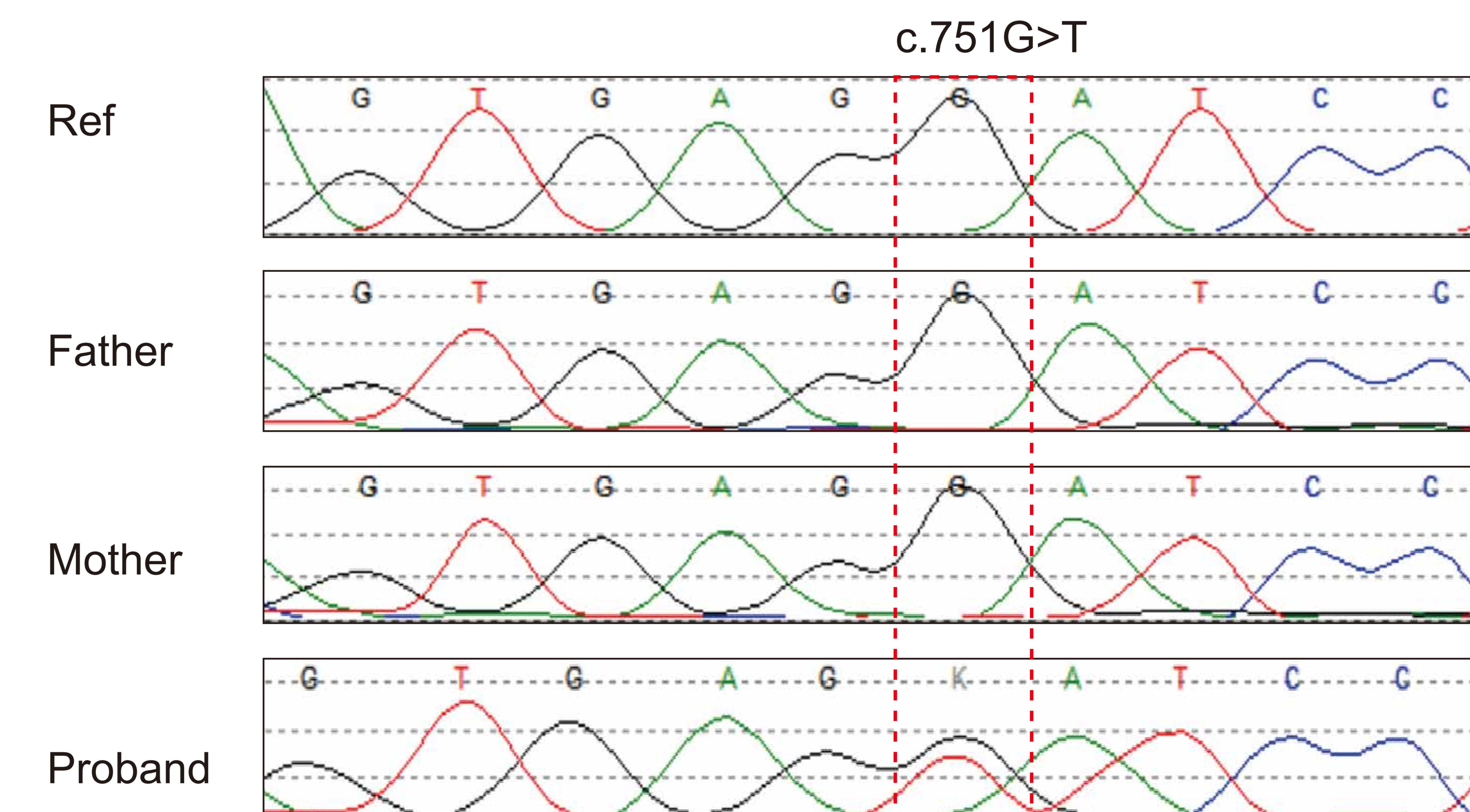

Supplement: Supplementary file 1 [file children-11-00897-s001.zip › Supplementary Figure S1.pdf]

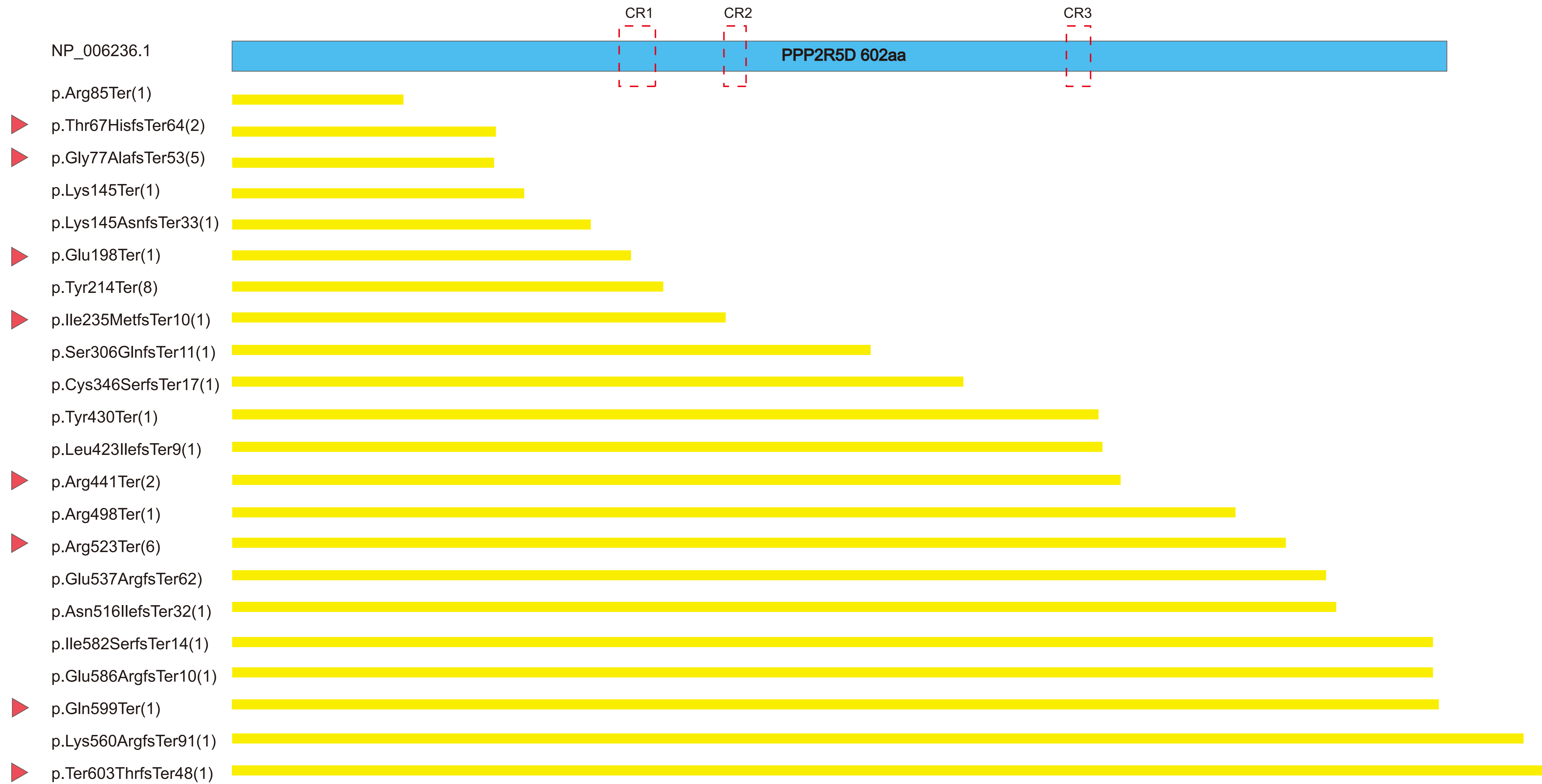

Supplement: Supplementary file 1 [file children-11-00897-s001.zip › supplementary Figure S3.pdf]
